# The Beer Question

**Published:** 1900-12-22

**Authors:** 


### The Beer Question.

The continued occurrence of cases of injury and even of death attributable to the consumption of arsenical beer raises many questions which will require most careful consideration. Accepting it as proven that a large proportion of the sufferers from arsenical poisoning of whom we have lately heard so much have derived their arsenic from beer, and that the arsenic obtained access to the beer by way of certain materials employed by the brewers in place of malt, we could quite sympathise with the cry which has been raised in favour of legislation which shall render it compulsory upon the brewers to put nothing into their beer but malt and hops—if that were all. It seems so simple; it seems so obvious that if nothing but pure malt and pure hops were put in, nothing but pure beer could come out. But we are not sure that this expresses the whole case, or that this is the best or only way. We remember the olden days when perhaps beer was pure; but it was not always nice. The strong ales, brewed at the proper time of year and kept for months, were undoubtedly very good, and so they are now; but the ordinary beer was very different stuff from the brilliant light ales which the confessedly substitute-using brewers now give us, and although it is quite probable that if the same amount of care and thought had been devoted to the production of a cheap light ale from malt as has been given to the production and use of malt substitutes a far better result might have been attained, the fact remains that the brewers of exclusively malt beer do not seem so far to have been able to provide the cheap beer of the quality that is asked for. Hence the problem is largely mixed up with the question of price, and as the provision of a sound, clear, and only slightly alcoholic beer seems to us the principal means at our disposal with which to withstand the tide of whiskey which is flowing over the land, and which is, without doubt, the first cause of many of those traits of modern life which indicate the oncoming of national decrepitude, we cannot regard the more or less plausible demand for a return to "malt and hops" as a true solution of the problem, unless it can be shown that with modern appliances a beer can be brewed which is cheap and light as well as pure.

And this is the root of the question. As for purity *of course* beer ought to be pure, that goes without saying, and so ought the materials from which beer is brewed, whether these consist of malt or glucose or maltose, or any other substance from which the brewer finds that he can make the best and cheapest beer. It cannot be denied that a large proportion of the substitute-brewed beer is perfectly good, and is, in fact, indistinguishable from beer brewed from the purest of malts. The present trouble never could have arisen unless the use of contaminated materials had been allowed in the brewing. The error, nay, we would say the crime, has been the selling and the using of a poisonous compound in the preparation of an article of diet, and if it can be shown that the introduction of such poisonous compounds cannot otherwise be prevented, then by all means let us go back to malt beer; but such a conclusion would be a miserable confession in these days of science. We do not think, however, that so far this has been shown to be the case; indeed, we do not know that any serious attempt even has been made to supervise or control the materials used in brewing so far as their chemical purity is concerned, and unless it can be shown that this has been tried and has failed, the entire prohibition of the use of anything but malt seems a somewhat clumsy device. Beer ought to possess certain qualities; among these, one at least should be freedom from poision; and the man who sells poison-containing beer ought to be severely punished. That seems to be the end of the matter. For Parliament to intervene regarding the recipe from which the beer is to be brewed is a measure which may possibly be necessary. But it is an extreme measure; one which involves a degree of scepticism as to the power of chemistry which is quite contrary to the drift of modern times; one, the necessity of which has certainly not yet been shown; and one, which, before its adoption, requires to be considered in all its bearings including the price, the quality, and the purity of the final product which is sold to the public—matters far more important than the mere restriction of the brewer to one form of fermentable material. Let us suggest a problem. Suppose—and in these days of advanced and "scientific" farming it is quite a reasonable supposition—that it were found possible to prevent mildew and smut, or any of the other maladies to which barley is subject, by spraying the growing corn with a weak arsenical solution, or with nicotine, or with some other of the hundred-and-one poisons at the disposal of the agricultural chemist; how would the drinker of "pure beer"—pure malt-and-hop-beer—fare then? The trust in malt and hops must always be but a broken reed unless proper care and supervision be exercised in regard to the materials going into the mash tub, and if this be granted, malt and its substitutes are on all fours with one another. In reference to this point attention must be drawn to the warning given by Mr. Estcourt, analyst to the city of Manchester, to the effect that he has found arsenic in several makes of malt, and in sufficient quantity to show in the beer produced from it as much arsenic as has been found in beer brewed from arsenical glucose. Nothing can get over the fact that, in regard to beer, as in regard to every other article of food, the protection of the public lies not in minute interferences with the processes of production, but in imposing the severest penalties on those who foist the poisoned or the adulterated stuff upon the consumer.

